# Modified Electrodes Used for Electrochemical Detection of Metal Ions in Environmental Analysis

**DOI:** 10.3390/bios5020241

**Published:** 2015-04-29

**Authors:** Gregory March, Tuan Dung Nguyen, Benoit Piro

**Affiliations:** 1 Klearia, route de Nozay, Marcoussis 91460, France; E-Mail: gregory.march@free.fr; 2 Institute for Tropical Technology, Vietnam Academy of Science and Technology, 18 Hoang Quoc Viet, Cau Giay District, Hanoi, Vietnam; E-Mail: ndung@itt.vast.vn; 3 Chemistry Department, University Paris Diderot, Sorbonne Paris Cité, ITODYS, UMR 7086 CNRS, 15 rue J-A de Baïf, 75205 Paris Cedex 13, France

**Keywords:** heavy metals, electrochemical sensors, stripping voltammetry, conducting polymers, DNA, enzymes, whole cells

## Abstract

Heavy metal pollution is one of the most serious environmental problems, and regulations are becoming stricter. Many efforts have been made to develop sensors for monitoring heavy metals in the environment. This review aims at presenting the different label-free strategies used to develop electrochemical sensors for the detection of heavy metals such as lead, cadmium, mercury, arsenic *etc.* The first part of this review will be dedicated to stripping voltammetry techniques, on unmodified electrodes (mercury, bismuth or noble metals in the bulk form), or electrodes modified at their surface by nanoparticles, nanostructures (CNT, graphene) or other innovative materials such as boron-doped diamond. The second part will be dedicated to chemically modified electrodes especially those with conducting polymers. The last part of this review will focus on bio-modified electrodes. Special attention will be paid to strategies using biomolecules (DNA, peptide or proteins), enzymes or whole cells.

## 1. Introduction

Heavy metals (HMs) are persistent in the environment (waters and soils), which means that they cannot be degraded. HMs mainly come from anthropic activities such as mining, smelting, or different kinds of wastes. Keep in mind that, among HMs, although some are necessary for life (iron, selenium, cobalt, copper, manganese, molybdenum, zinc), unfortunately, many others are toxic. For example, mercury (Hg) enters the environment through not only coal burning but also through mining or industrial wastes, and is known to cause damage mainly to the nervous system; Lead (Pb) comes from automobile exhausts, old paints, mining wastes, incinerator ash or water from lead pipes and is also known to causes damage to the nervous system; Cadmium (Cd), which causes kidney diseases, comes from the electroplating and mining industries; lastly, arsenic (As) comes from herbicides or, again, from the mining industry and causes damage to skin, eyes, and liver. Other major heavy metals are chromium, nickel, tin and thallium. For sanitary reasons, it has become necessary to detect and quantify HMs in soils or in waters. For example, French regulations set mercury contents in drinking water at 1 µg·L^−1^, silver or lead at 10 µg·L^−1^ and arsenic at 50 µg·L^−1^.

Traditional analytical methods are atomic absorption or emission spectroscopies (AAS, AES [1,2]), inductively coupled plasma mass spectrometry (ICP-MS [3]) or cold vapor atomic fluorescence spectrometry (CVAFS [4]). They are extremely sensitive but are however expensive and require laborious pre-treatment processes [5]. Therefore, in some cases, these methods may be replaced by more easy-to-use and inexpensive ones such as sensors, which may be based on either optical transduction (e.g., photonic crystal sensors [6,7,8,9]) or on electrochemical transduction (ion-selective electrodes, polarography, potentiometry, amperometry, conductimetry…) on which the review will more specifically focus. Other transduction methods have been extensively reviewed elsewhere.

Heavy metals are generally present under their cationic form (Hg^2+^, Pb^2+^, Cd^2+^…), therefore, they can be electroreduced to the corresponding metal at an electrode; this corresponds with a preconcentration step, because a large amount of metal can be deposited on the electrode even if the cation concentration is low, providing that the deposition (electroreduction) time is sufficiently long. In a second step, an anodic potential scan is applied, so that the metal is oxidized back to the corresponding cation. This electrochemical reaction is extremely fast, giving a strong current (proportional to the quantity of metal ion initially present in the medium) and providing high sensitivity; in addition, each metal is oxidized at a particular potential, which provides specificity. This method is called Anodic Stripping Voltammetry (ASV) and is extremely pertinent for HMs quantification. The first part of this review will be dedicated to this technique used on unmodified electrodes (mercury, bismuth or noble metals in the bulk form), or electrodes modified at their surface by nanoparticles, nanostructures (CNT, graphene) or other innovative materials such as boron-doped diamond.

Another way to functionalize electrodes is to use conducting polymers (polypyrrole, polythiophene, polyaniline, polynapthalene, *etc*.). One main reason for the interest in conducting polymers (CPs) is that small perturbations at their surface or in their bulk can generate strong changes in their electroactivity, which can be probed by amperometry or potentiometry. Another interest of CPs, beyond the simplicity they offer in modifying a conducting surface, is that they are easy to functionalize with additional chemical functions. The easiest way is to dope CPs with doping ions carrying the required function. The other way is to chemically modify the backbone of the CPs with the desired function. Additionally, it has been shown that some CPs are biocompatible, and therefore are convenient for binding biomolecules.

Biomolecules such as DNA, peptides or proteins have been reported to selectively bind HM ions. This allows for selective preconcentration of ions at an electrode surface before stripping voltammetry, therefore avoiding interferences between metals. Lastly, beyond the use of simple biomolecules, enzymes (generally phosphatase or urease) have been also employed for the detection of HMs, which interact with their active site and lower their activity (this phenomenon is called enzyme inhibition). However, isolated enzymes immobilized on electrodes are often unstable; this is why whole cells such as microalgae or other micro-organisms, containing themselves active enzymes, have attracted much interest. All these items will be detailed in this review.

## 2. Generalities on Stripping Voltammetry

**Figure 1:**
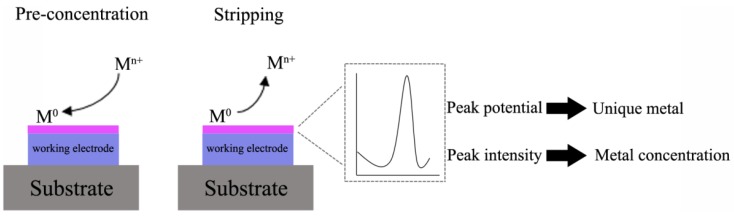
Anodic Stripping Voltammetry (ASV) principle.

Stripping voltammetry comprises a group of various techniques including Anodic Stripping Voltammetry, Cathodic Stripping Voltammetry (CSV) and Adsorptive Stripping Voltammetry (AdSV). It is an ultrasensitive detection technique based on electrochemical measurements similar to polarography. Stripping voltammetry is a two-step technique that allows simultaneous detection of various inorganic and organic species in the sub-nanomolar range. The first step consists of the electrolytic deposition of a chemical species onto an inert electrode surface at a constant potential. This preconcentration step explains the remarkable sensitivity of the technique. It can involve either an anodic or cathodic process. However, the most common use of stripping voltammetry involves a cathodic process for deposition in which the metal ionic species are reduced from the solution to the electrode surface. The second step consists of the application of a voltage scan to the electrode. At a specific potential, it causes the stripping of a specific species accumulated onto the electrode surface as amalgam or thin films, into the solution. The resulting faradic current is proportional to the concentration of the chemical species (Figure 1).

In AdSV, conversely to ASV, the preconcentration step of the metal ions at the surface of the electrode is made by a non-electrochemical process, in the form of complexes. Notably, Ni and Co were quantified by AdSV after complexation with dimethylglyoxime [10], Cr after complexation with cupferron [11] and Al after complexation with cupferron [12] or Eriochrome blue black R [13]. In analytical practice, stripping voltammograms are recorded in the Differential Pulse (DP) or Square Wave (SW) modes, which are generally more sensitive than normal scan mode. Table 1 addresses a list of HMs detectable by these three different stripping voltammetries.

**Table 1:** List of heavy metals that can be detected with stripping voltammetry techniques.

**Metals that can be determined by Anodic Stripping Voltammetry**		
Antimony	Gallium	Mercury
Arsenic	Germanium	Silver
Bismuth	Manganese	Thallium
Cadmium	Indium	Tin
Copper	Lead	Zinc
**Species that can be determined by Cathodic Stripping Voltammetry**		
Arsenic	Iodide	Mercaptans
Chloride	Selenium	Thiocyanate
Bromide	Sulfide	Thio compounds
**Metals that can be determined by Adsorptive Stripping Voltammetry**		
Aluminum	Nickel	Uranium
Cobalt	Chromium	Iron

## 3. Electrode Materials

### 3.1. Mercury Electrodes

The performance of ASV is strongly influenced by the material of the working electrode. Traditionally, hanging mercury drop electrodes (HMDE) have been used, mainly because clean surfaces can be easily regenerated with a new mercury drop. Moreover, the potential window where mercury stays electro-inactive is very large, so that very electronegative metals can be detected. Despite being long known as an electrode, it is still used and upgraded. For example, Adam *et al.* [14] have detected Cd^2+^ and Zn^2+^ with a phytochelatin-modified HMDE using AdSV. However, HMDE present several drawbacks: metallic ions such as Hg, Au and Ag cannot be measured and the use of mercury electrodes is now severely restricted due to obvious toxicity considerations. Mercury thin film electrodes (MFEs) could be an alternative as less mercury is necessary. Nevertheless, the development of mercury-free analytical systems is becoming inevitable.

### 3.2. Gold and Silver Electrodes

Kirowa-Eisner *et al.* used gold [15,16] and silver [17,18,19] electrodes to detect cadmium, lead and copper. They showed that gold is unsuitable for mixtures of lead and cadmium because of overlapping of the two stripping peaks. Silver exhibits excellent characteristics for lead and cadmium detection: high repeatability and long-term stability without the need of any pretreatment, with limits of detection (LoD) in the nM range. The sensitivity can be further improved by using advanced procedures or electrode surfaces. For example, Compton’s group has detected arsenic(III) with a LoD of 1 ppb on gold [20] and silver [21] electrodes using ASV assisted with ultrasound. Rahman *et al.* [22] have reached a LoD of 0.28 ppb using Au(111)-like polycrystalline electrodes. They have shown that trace level of As(III) could be detected in tap water even in the presence of Cu. Total inorganic arsenic determination was achieved using differential pulse ASV (DPASV) in real samples at a gold-coated diamond thin film electrode with a LoD of ca. 20 ppb [23]. Note that Compton *et al.* have also shown that gold electrodes are highly sensitive for the detection of chromium(VI) even with cyclic voltammetry, with a LoD of 228 ppb and have proven superior performances compared to glassy carbon and bore doped diamond [24]. Using gold film-modified carbon composite electrode, Kachoosangi and Compton achieved a LoD of 4.4 ppb for chromium(VI) [25].

### 3.3. Gold Nanoparticles-Modified Electrodes

**Figure 2:**
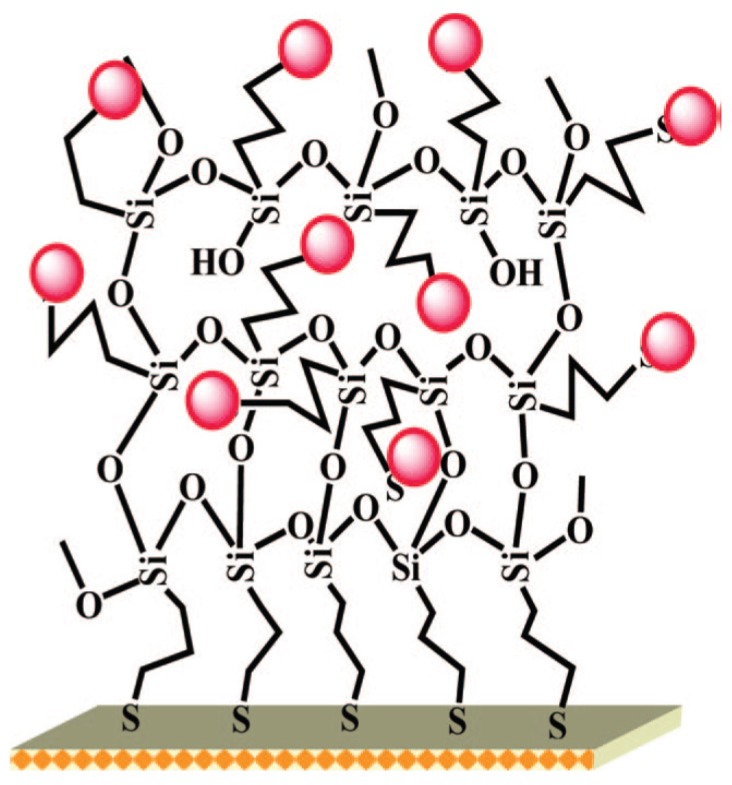
Schematic illustrations of gold nanoelectrode ensembles (GNEEs) dedicated to the detection of arsenic using a colloidal chemical approach on thiol-functionalized 3D silicate network preassembled on polycrystalline gold electrode. Reprinted with permission from [27]. Copyright 2008 American Chemical Society.

Nanostructured gold electrodes have been shown to improve LoD. In particular, several papers described the successful use of gold nanoparticles-modified electrodes. Compton’s group developed a gold-NPs modified glassy carbon electrode for arsenic(III) detection, with a LoD of 0.0096 ppb using LSV [26]. Jena *et al.* developed a highly sensitive platform based on gold nanoelectrodes ensembles (GNEEs). GNEEs were grown by colloidal approach on thiol-functionalized 3D silicate network preassembled on polycrystalline gold electrode (Figure 2). Using square wave anodic stripping voltammetry (SWASV), they achieved simultaneous detection of arsenic(III) and Hg(II) in presence of Cu(II) with a LoD of 0.02 ppb [27]. Detection of Cr(VI) was also achieved with the same platform with a LoD of 0.1 ppb [28]. Mordegan *et al.* developed GNEEs using a polycarbonate membrane as template for a better control of the nanoelectrodes density. A LoD of 5 ppt was achieved for arsenic detection [29].

### 3.4. Bismuth Film Electrodes

In 2000, bismuth-film electrodes (BFEs) were introduced as an alternative to mercury film electrodes (MFEs) [30]. BFEs are prepared by plating thin bismuth films on suitable electrode materials. The main advantages of the BFEs are that they are environmentally friendly, since the toxicity of bismuth and bismuth ions is negligible, and their analytical properties are comparable to those of MFEs. Bismuth can be plated on the same substrate as mercury: glassy carbon [31], screen-printed carbon ink [32,33] and gold [34] have been successfully used. It has also been shown that BFEs offer a better separation between intermetallic compounds than MFEs, e.g., Cd^2+^ and Pb^2+^, even if a large excess of Cu^2+^ is present [35,36].

The method for electroplating the electrode surface with bismuth is critical to obtaining satisfactory performances. Three methods exist to realize a bismuth deposit [37]. The first method, *ex situ* plating, consists of electroplating the bismuth film before transferring the electrode into the sample solution for analysis. The plating conditions are variable but acidic media are recommended as bismuth ions are easily hydrolyzed as high pH. Plating solutions containing 5–200 mg·L^−1^ Bi(III) are used with a deposition potential comprised between −0.5 and −1.2 V and deposition time of 1–8 min under conditions of forced convection (electrode rotation or convection). The second method, *in situ* plating, consists of adding directly Bi(III) ions in the concentration range 400–1000 mg·L^−1^ into the sample solution. The bismuth film is deposited onto the electrode surface during the analysis. The main restriction of this method is the pH which must be fairly acidic, for the reason given above. The last method is essentially confined to carbon-paste electrodes. It is based on modifying the bulk of an electrode with bismuth compounds such as Bi_2_O_3_.

*Ex situ* plating is more versatile as electroplating conditions are independent of analysis conditions but more complex and time consuming than *in situ* plating. Moreover, BFEs prepared with the *ex situ* method can be reused: the surface of the electrode is reactivated after each analysis by polarization at an adequate potential, more negative than the oxidation potential of bismuth but more positive than the oxidation stripping potential of metal ions. In practice, a short cleaning step of 10–30 s at −0.35 V to –0.40 V in stirred solution is sufficient. For the *in situ* method, the bismuth film is stripped after each analysis at a potential more positive than the oxidation potential of bismuth, typically in the range 0.0 V to +0.3 V, during a few tens of seconds, then a new bismuth film is re-plated.

**Figure 3:**
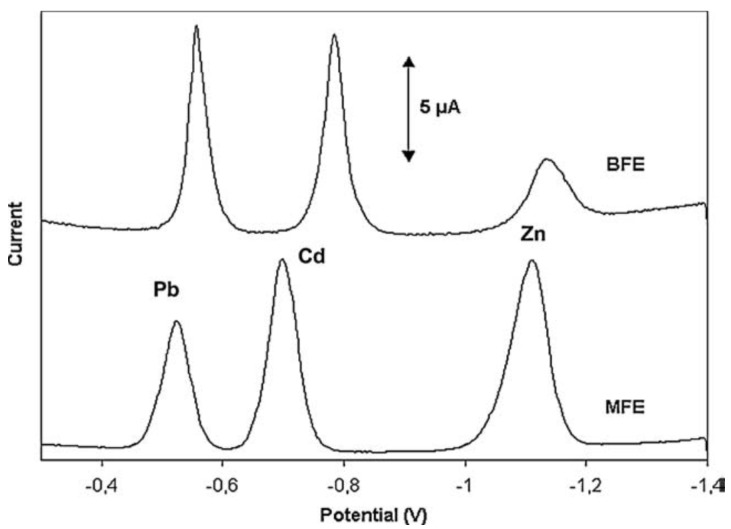
Comparative voltammograms of a solution containing 20 ppb each of Pb(II), Cd(II) and Zn(II) in 0.1 M acetate buffer (pH 4.5) on *in situ* plated BFE and MFE on a glassy carbon support. Reprinted from [37] with permission from Elsevier.

The main disadvantage of BFEs compared to MFEs is their lower potential window, particularly a more negative anodic limit due to the fact that bismuth is more easily oxidized than mercury. However, the cathodic limit potential is almost the same as MFEs. The pH of the sample solution strongly affects the useful potential window of BFEs. As expected, the most cathodic potential limit was achieved in basic media whereas the most anodic potential limit was achieved in very acidic media. Table 2 shows potential ranges of BFEs at different pH values [37], and Figure 3 shows an example for simultaneous Pb^2+^, Zn^2+^ and Cd^2+^ determination.

**Table 2:** Potential ranges of BFEs plated on carbon paste at different pH values (numerical values taken from [37]).

Medium	pH	Anodic Limit (V)	Cathodic Limit (V)	Potential Window (V)
**0.1M HClO_4_**	1.00	−0.05	−1.05	1.10
**0.2M acetate buffer**	4.24	−0.25	−1.25	1.00
**0.1 M NaOH**	12.17	−0.55	−1.55	1.00

Nunes *et al.* [38] studied the influence of electrodeposition conditions on the electroanalytical performances of bismuth films deposited on copper electrodes. They showed that the morphology of the film was of great importance, the most homogeneous films giving better analytical performances for lead detection. Evaporated bismuth was also used, which provided favorable reproducibility and sensitivity for heavy metal detection [39,40] and could be easily shaped into arrays [41,42] or integrated into microfluidic devices [43].

### 3.5. Antimony Film Electrodes

**Figure 4:**
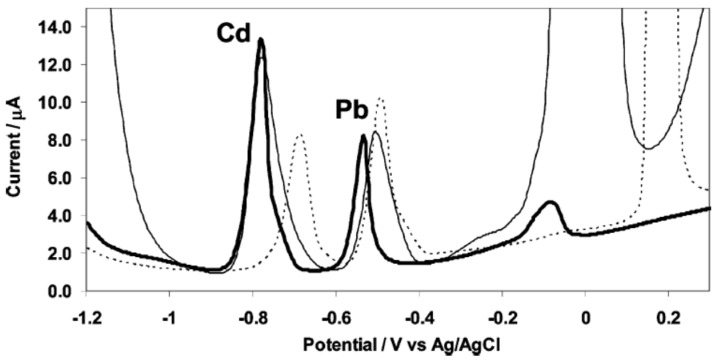
Anodic stripping voltammograms of cadmium(II) and lead(II) at *in situ* prepared antimony film (thick line), bismuth film (thin line) and mercury film (dashed line) electrodes. Solution: 0.01 M hydrochloric acid (pH 2) containing 50 ppb cadmium(II) and lead(II) together with 1 mg·L^−1^ antimony(III), bismuth(III), or mercury(II). Reprinted with permission from [44]. Copyright 2007 American Chemical Society.

The use of antimony film electrodes (SbFEs) for electrochemical stripping analysis was first reported by Hocevar *et al.* in 2007 [44] (Figure 4) although the fact that the use of antimony as electrode material was known since 1923 for pH measurements [45]. As for bismuth, two methods of plating are commonly used: *ex situ* and *in situ* plating. SbFEs exhibit a very similar electrochemical stripping behavior to BFEs, with almost the same sensitivity towards Pb(II) and a slightly higher sensitivity towards Cd(II). With respect to the MFEs, both BFEs and SbFEs provide an improved sensitivity towards cadmium whereas it is the opposite for lead. Also, compared to BFEs, SbFEs provides a more favorable hydrogen evolution which is comparable to MFEs. SbFEs were notably used for detection of Pb, Cd and Zn in tap and river water [46] and for the detection of Ni using complexation with dimethylglyoxime [47,48].

### 3.6. Bore Doped Diamond (BDD)

Conductive diamond films are a new type of material which are used more and more for electroanalytical purpose. Indeed, these materials present remarkable properties among carbon materials: (i) a low and stable background current; (ii) relatively high electronic transfer rate; (iii) a low molecular adsorption; (iv) resistance to corrosion; and (v) optical transparency. Usually, diamond films are deposited on conductive substrates such as highly doped silicon wafer, molybdenum, tungsten or titanium using plasma-wave assisted chemical vapor deposition (PACVD) or hot filament.

At the pristine state, diamond is one the best known electrical isolators. In order to have an appropriate conductivity to be used as an electrode for electrochemical measurements (>10 S·cm^−1^), diamond needs to be doped. The most frequently used dopant is boron and such electrodes are called boron doped diamond (BDD). BDD film can reach resistivity of <0.05 Ω·cm.

**Figure 5:**
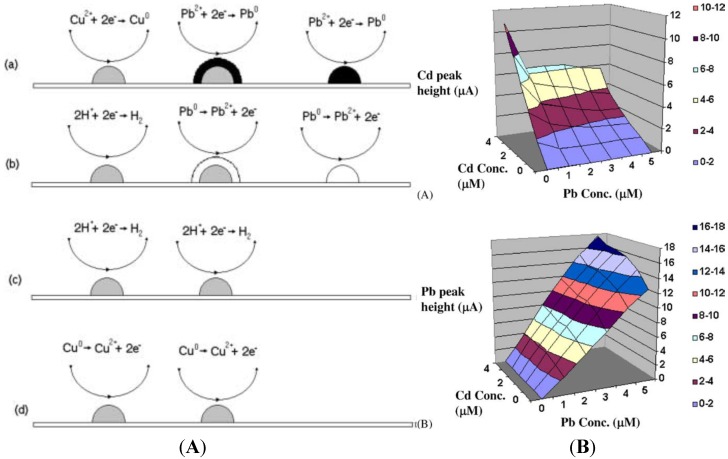
(**A**) Schematic diagram for the deposition and stripping processes. (a) The potential is maintained at −1.0 V where lead and copper deposits are formed but the latter are quickly covered by reduced lead. (b) When scanning in the positive direction, lead is first oxidized, (c) leaving copper deposits exposed, and hydrogen evolution takes place. (d) Continuing scanning at potentials E > 0.0 V Cu deposits are oxidized; (**B**) Three-dimensional calibration curves for the differential pulse anodic stripping currents for (a) Cd (II) (b) Pb (II) in acetate buffer solutions containing both metals. Reprinted from [64], with permission from Elsevier.

The reason why BDD electrodes attract much attention for stripping analysis of heavy metals is that they can be used within a very wide potential window. Peilin *et al.* [49] demonstrated that BDD has a slightly better sensitivity for lead (3 nA·mm^−2^·ppb^−1^) than glassy carbon electrode (GCE) (2.4 nA·mm^−2^·ppb^−1^), when both use *in situ* plated mercury. However, BDD without mercury plating exhibits three to five times lower sensitivity compared to mercury plated GCE [50]. With ASV, Manivannan *et al.* have reported that sub-ppb detection of lead is achievable using −1 V deposition potential for 15 min [51,52,53]. Compton’s group has shown that sonoelectrochemical treatment increased sensitivity for Pb [54], Cd [55], Mn [56] and Ag [57]. BDD electrodes have been successfully used for the simultaneous detection of mixtures of HMs: Pb + Cd + Ag [58], Zn + Pb + Cd + Cu [59], Pb + Cd + Cu + Hg [60] and Cd + Ni + Pb + Hg [61]. Hutton *et al.* have examined the factors controlling stripping voltammetry of lead at BDD using high-resolution microscopy [62]. They have shown that the deposition process was driven to produce a grain-independent homogeneous distribution of Pb nanoparticles on the electrode and that substantial amount of Pb remains on the surface after stripping, which explains the non-linear response at high concentrations. Prado *et al.* studied the interaction between Pb and Cu [63] during simultaneous detection by ASV. They observed the appearance of an extra peak which was attributed to hydrogen evolution on copper. They suggested that Cu deposition occurs preferentially, then Pb deposition takes place on already formed copper deposits which act as active sites for nucleation and growth process. This covers the copper with a solid film of lead. During the stripping step, oxidation of lead takes place first, so the copper deposits would be suddenly exposed to an acid electrolyte at a potential at which hydrogen evolution could take place. Manivannan *et al.* [64] studied the interaction between Pb and Cd during simultaneous detection (Figure 5A). They observed that in the presence of a constant concentration of Pb (5 µM), the peak currents for Cd were ca. 55% smaller than those obtained without Pb. On the contrary, they also observed that in the presence of a constant concentration of Cd (5 µM), the peak currents for Pb were ca. 40% larger compared to those for Pb in the absence of Cd. This behavior was explained through the model described above for Pb and Cu, *i.e*., metals that have more negative standard potentials tend to deposit on metals that have less negative standard potentials. This explains the difference in peak current observed for Cd and Pb. The authors proposed a 3D calibration curve to avoid cross-interference between these two metals (Figure 5B).

### 3.7. Diamond Like Carbon

The use of BDD as electrode materials generates a great interest in the field of amorphous carbon or “diamond like carbon” (DLC) for electrochemical purpose. DLC films have electrochemical properties similar to those of BDD: low background current, wide potential window and high electronic transfer rate. Unlike BDD, DLC films exhibit the main advantage in being able to be synthesized at low temperatures (from ambient temperature to 200 °C), so they can for example be integrated to glass microfluidic devices, for example. Amorphous carbon films have a microstructure which is a mix of sp^2^ and sp^3^ carbon. They can also contain hydrogen, depending on deposition conditions. A wide range of microstructures can be obtained with different sp^2^/sp^3^ carbon ratio and hydrogen content. Films with high level of sp^3^ carbon (>40%) are called tetrahedral amorphous carbon (Ta-C). Various elaboration methods can be used: (i) ion deposition; (ii) filtered cathodic vacuum arc (FCVA); (iii) pulsed laser deposition which lead mainly to Ta-C films; (iv) sputtering; (v) plasma enhanced chemical vapor deposition (PECVD) which lead to amorphous carbon films containing higher level of sp^2^ carbons [65]. Zeng *et al.* [66] used sputtered DLC film for simultaneous detection of Pb, Cd and Cu. Khun *et al.* [67] and Liu *et al.* [68] used tetrahedral nitrogen doped amorphous carbon (Ta-C:N) for the detection of single elements like Zn, Cu, Pb and Hg and simultaneous detection of Pb, Cu and Hg in deaerated and unstirred KCl solution, but the concentrations used were high (mM). Khadro *et al.* [69] used femto laser ablation to deposit undoped a-C and doped a-C:B (8%) film onto SiO_2_ and Si_3_N_4_ substrates. Pb, Ni, Cd and Hg were detected using SWASV. The effect of the boron doping was clearly evidenced for Pb detection: The Pb peak height increased by 20% with 8% of bore as dopant.

As a conclusion of this section dedicated to electrode materials, it has been proved that stripping voltammetry on metallic or carbon electrodes is very effective in detecting various heavy metals at sub-ppb levels even in real samples such as tap water. Such systems are also easily miniaturisable for on-field detection. Nevertheless, they present important drawbacks, such as in the exact determination of a concentration in presence of mixture of heavy metals, lack of reproducibility due to intermetallic compounds, to fouling of the electrode after measurements and the formation of biofilms. One solution to these drawbacks is to chemically modify the electrode surface in order to enhance selectivity and sensitivity or to prevent fouling; conducting polymers are excellent candidates for such electrode modifications.

## 4. Conducting Polymer-Modified Electrodes

Chemically modified electrodes (CMEs) have received increasing attention, because they enhance sensitivity and selectivity of electrochemical analysis techniques. Among various modification approaches, conducting polymers (CPs) have received considerable attention due to their superior electrical conductivities, good adhesion properties and easy preparation. In addition, CPs demonstrated also anti-fouling capability, which is an important practical advantage over conventional electrode materials.

### 4.1. Unmodified Conducting Polymers

**Figure 6:**
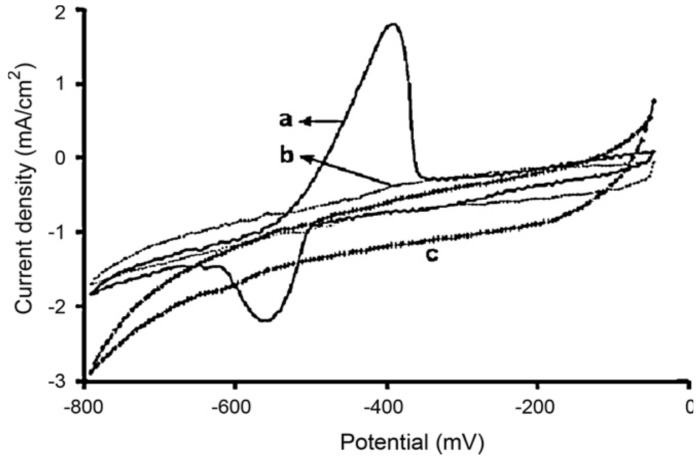
Cyclic voltammograms for 1 mM Pb^2+^ in 0.05 mol·L^−1^ HCl on: (**a**) PEDOT: PSS-modified electrode and (**b**) bare carbon electrode, compared with (**c**) lead-free electrolyte on PEDOT:PSS-modified electrode. Potentials *vs*. Ag/AgCl. Scan rate 10 mV·s^−1^. Reprinted from [75], with permission from Elsevier.

Unmodified CP films may display intrinsic affinity to metal ions. For example, it was demonstrated that silver could be entrapped in polypyrrole film [70,71]. Polythiophene and its derivatives also show affinity with HMs. Zejli *et al.* electropolymerized poly(3-methylthiophene) (P3MT) on gold or sonogel electrodes [72,73]. Mercury(II) was preconcentrated from the solution into the P3MT-modified electrode at open-circuit potential, then differential pulse voltammetry (DPV) was used to reoxidize Hg^0^; a linear dependence with mercury(II) concentration was obtained in the range 10^−8^–4 × 10^−6^ mol·L^−1^. Poly(3,4-ethylenedioxythiophene): polystyrenesulfonate (PEDOT:PSS) was also used towards lead ions detection [74] (Figure 6).

Recently, Liu *et al.* demonstrated a highly Ag^+^-sensitive electrochemical sensor made of GCE coated with a Langmuir-Blodgett film (LB) that contained polyaniline (PANi) doped with p-toluenesulfonic acid (PTSA) [75]. An advantage of CPs over bare GC electrodes was also reported by Wang *et al*. [76], who evidenced that the quantity of metal remaining on the surface of PANi/GCE was smaller than on the bare GCE after stripping, which improved the repeatability of the electrodes. In comparison with typical CPs such as PPy, PANi and PTh, CPs from aromatic diamines have lower electrical conductivity, but they have a unique ability to form stable complexes with HM ions. Many references reported that polydiaminonaphthalene (PDAN) could chelate Cu^2+^ [77,78], Hg^2+^, Ag^+^ [79,80], Se(IV) [81] or Pb^2+^, probably via free -NH_2_ groups. For example, Majid *et al.* [82] used a poly(1,8-diaminonaphthalene)-modified electrode to detect Pb^2+^ by ASV, with a preconcentration step at a potential of −0.9 V *vs*. Ag/AgCl before anodic stripping in differential pulse mode. For a preconcentration time of 10 min, they obtained a linear calibration from 4 pg·L^−1^ to 2 ng·L^−1^.

### 4.2. Modified Conducting Polymers

As shown above, some CPs present intrinsic properties which may confer sensitivity and selectivity towards HMs; however, to improve these properties, they must be functionalized with ligands, for example, by making copolymers, by entrapment of known ionophores or using selected counter ions.

#### 4.2.1. Doping and Copolymerization

**Figure 7:**
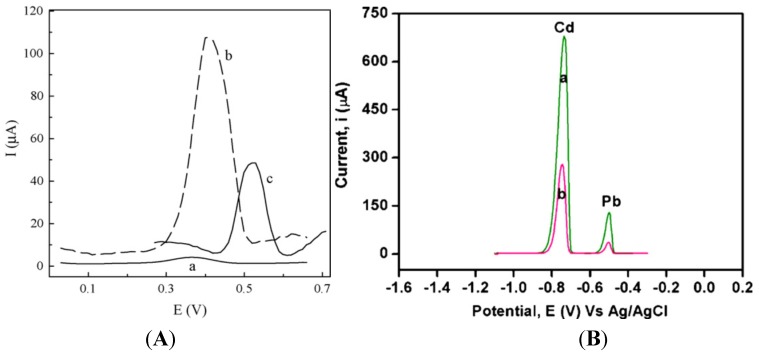
(**A**) DPASV response for a bare GC electrode (a) and for a EBB/PPy-modified electrode (b) after preconcentration in 10^−5^ M Ag^+^. The response of the electrode to 10^−5^ M Hg^2+^ is also shown in (c). Reprinted from [83] with permission from Elsevier; (**B**) DPASV of the solution containing Cd^2+^ (12.8 ppm) and Pb^2+^ (16.6 ppm) recorded at (a) a poly(diphenylamine-co-2-aminobenzonitrile)-modified electrode and (b) poly(diphenylamine)-modified electrode. Deposition potential = −1.0 V; deposition time = 60 s; pH = 2.1. Reprinted from [88] with permission from Elsevier.

The use of functional doping ions is probably the simplest way to functionalize CPs. For example, PPy membranes were deposited on GCE by electropolymerization of pyrrole in the presence of Eriochrome Blue-Black B (EBB) as counter anion [83]. The differential pulse anodic stripping voltammetry (DPASV) response of the EBB/PPy-modified electrode *versus* a bare GCE, both after preconcentration in 10^−5^ M Ag^+^ at −0.4 V for 200 s, is shown in Figure 7A; the sensor showed a LoD of ca. 6 × 10^−9^ M for Ag^+^. Following the same idea, Lisak *et al.* described a polybenzopyrene film into which Eriochrome black T was entrapped, able to form complexes with Pb^2+^ ion [84]. In another study, a polythiophene-quinoline (PTQ)-modified electrode was used to detect copper and mercury. The redox behaviors of Cu(II) and Hg(II) were almost identical on this electrode, but the addition of 4-(2-pyridylazo)resorcinol (PAR) allowed the separation of the two cations due to the formation of a Cu(II)-PAR complex reduced at −0.8 V, whereas that of Hg(II) appeared at −0.5 V *vs*. SCE. Hg(II) was detected down to 0.4 ppb [85].

**Figure 8:**
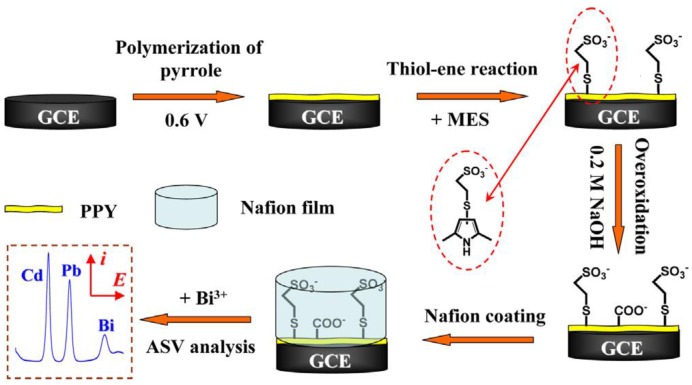
Schematic illustration of the procedures for preparing the Bi/Nafion/OPPy-MES/GCE for ASV analysis of Cd^2+^ and Pb^2+^ ions (not to scale). Reprinted from [90] with permission from Elsevier.

Copolymerization is also a simple way to functionalize CPs. For example, Somerset *et al*. showed that the presence of Hg^2+^ can be determined with a polyaniline-methylene blue (PANi-MB) copolymer [86]. The electrodes were prepared by electropolymerization of a mixture of aniline and methylene blue. A linear calibration curve was found in the range of 10^−8^ M to 10^−5^ M Hg^2+^ using ASV. Copolymerization of ANi with 2,2'-dithiodianiline (DTDA) provided similar results [87]. In the case of Cd^2+^ and Pb^2+^, Philips *et al*. [88] developed a new copolymer poly(diphenylamine-co-2-aminobenzonitrile) and showed that it is efficient for detection of these ions down to 1.26 ppm and 0.26 ppm, respectively (Figure 7B). Copolymers of aniline and imidazole were also reported to present a high sensitivity toward Pb^2+^ (2 µg·L^−1^) [89]. In another interesting example, authors described an electrode modified with overoxidized 2-mercaptoethanesulfonate (MES)-tethered polypyrrole, Nafion and bismuth, by analogy with bismuth thin-film electrodes described in Section 3.4 of this review [90]. SWASV was applied to detect Pb^2+^ and Cd^2+^ with LoDs of 0.03 µg·L^−1^ and 0.04 µg·L^−1^, respectively (Figure 8).

**Figure 9:**
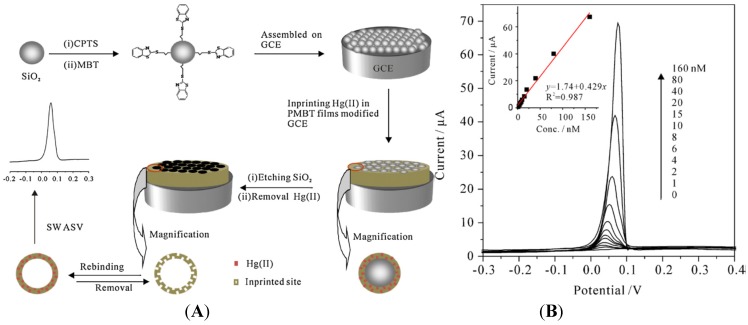
(**A**) Schematic representation of how Hg^2+^-imprinted MPMBT is obtained; (**B**) Typical SWASV of Hg^2+^ on an imprinted MPMBT. Preconcentration time: 8 min. Inset: calibration plot of the SWASV peak current *vs*. the Hg^2+^ concentration. Reprinted from [91] with permission from Elsevier.

Lastly, authors reported an innovative strategy using ion imprinting in electropolymerized poly(2-mercaptobenzothiazole) (MPMBT), for the detection of Hg^2+^ [91]. It consists of electropolymerization in presence of Hg^2+^, then removal of Hg^2+^ before the detection step (Figure 9A). SWASV on such Hg^2+^-imprinted MPMBT showed a LoD in the nM range (Figure 9B). They also showed that such electrode is insensitive to interferences for Pb^2+^, Cd^2+^, Zn^2+^, Cu^2+^ and Ag^+^.

#### 4.2.2. Grafting of a Complexing Agent

Another possibility of modifying CPs is to graft the complexing agent on the polymer. For example, Rahman *et al.* reported the electropolymerization of 3',4'-diamino-2,2';5',2''-terthiophene onto which ethylenediaminetetraacetic acid (EDTA) was covalently coupled. EDTA is able to complex Pb^2+^, Cu^2+^ and Hg^2+^ ions during the preconcentration step, which was followed by reduction at −0.9 V and reoxidation from −0.9 V to +0.75 V. Linear calibration plots using SWV were obtained from 5 × 10^−10^ M to 10^−7^ M for Cu^2+^ and from 7.5 × 10^−10^ M to 10^−7^ M for Pb^2+^ and Hg^2+^, with corresponding LoD of 6 × 10^−10^, 2 × 10^−10^ and 5 × 10^−10^ M, respectively. Covered by a Nafion film, these electrodes were stable for more than one month [92]. Another example of conducting polymer modified by a complexing agent was given by Heitzmann *et al*. Poly(*N*,*N*-ethylene*bis*[*N*-[(3-(pyrrole-1-yl)propyl) carbamoyl) methyl]-glycine]), noted poly**L**, which was used for the electrochemical detection of Cu^2+^, Pb^2+^ and Cd^2+^, following the same protocol as for the previous example. Experiments showed that the poly**L-**modified electrodes were selective towards Cu^2+^ and insensitive to Cd^2+^ [93].

Stripping voltammetry is not the only reported method. Cortina-Puig *et al.* described overoxidized pyrrole-modified electrodes carrying ionophores specific for K^+^, NH_4_^+^ for which electrochemical impedance spectroscopy was used for detection. The best results were obtained with dibenzo-18-crown-6 as ionophore [94]. Conductometry was also reported for detection of Hg^2+^ using cryptand-222 as the receptor immobilized on PANi, with a LoD of ca. 10^−12^ M [95]. As a last example, potentiometry was also used with polyaniline-modified electrodes onto which thiacalix[4]arene containing pyridine fragments was coupled, for determination of Ag^+^ ions, however with a poor LoD in the μM range [96].

#### 4.2.3. CNT-Modified Conducting Polymers

**Figure 10:**
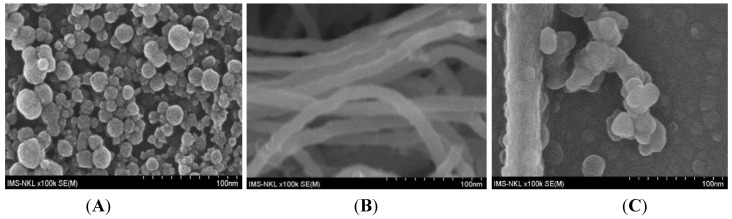
FE-SEM images of (**A**) PDAN; (**B**) pure CNT and (**C**) PDAN/CNT. Reprinted from [99] with permission from Elsevier.

Carbon nanotubes (CNTs) have a large surface-to-volume ratio, good conductivity and strong adsorption ability that were proved to be useful for improving the sensitivity of sensors in general, and ions sensors in particular. Applying these properties, Wang *et al.* [97] used PANi-modified CNTs to detect Pb^2+^ by SWASV and found that CNT:PANI-coated electrodes had better performances than bare GCE. Other publications demonstrated that CNTs functionalized with aromatic amines are efficient for HMs adsorption. For example, Salmanipour *et al.* [98] functionalized GC electrodes with a mixture of multi-walled carbon nanotubes (MWCNTs) and 2-(5-bromo-2-pyridylazo)-5-diethylaminophenol (5-Br-PADAP) and detected Pb^2+^ by ASV in a range from 1 to 115 μg L^−1^. CPs/CNTs nanocomposites may also be made electrochemically. For example, Nguyen *et al.* [99] electropolymerized a mixture of PDAN and CNT on interdigitated arrays (Figure 10).

**Figure 11:**
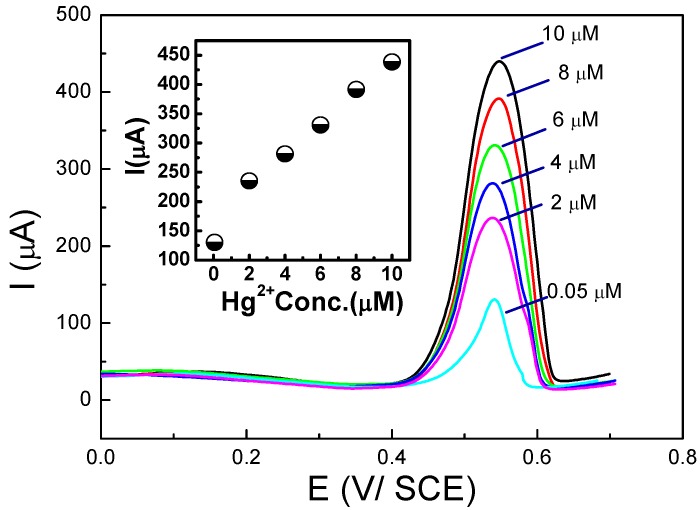
SWV curves, recorded on a PDAN/CNT-modified electrode, for various Hg^2+^ concentrations (0.05–10 µM) and corresponding calibration curve (inset). Reprinted from [99] with permission from Elsevier.

Detection of Hg^2+^ was performed through preconcentration at open circuit potential followed by SWV. This sensor was specific for mercury because of the Hg^2+^/Hg_2_^2+^ redox potential with respect to that of PDAN/CNT. Figure 11 presents the corresponding SWVs and calibration curve. The same group also demonstrated a new approach to fabricate CPs/CNTs hybrid using a mixture of Nafion^®^ (DuPont, Wilmington, DE, USA) and MWCNTs first deposited on the electrode surface, followed by 1,5-DAN electropolymerization [100,101], for determination of Cd^2+^ and Pb^2+^ by SWASV over the range 4–150 µg·L^−^^1^ and LoDs of 3.2 and 2.1 µg·L^−^^1^ for Cd^2+^ and Pb^2+^, respectively.

In conclusion, modification electrodes with CPs could improve their selectivity towards HMs and also their anti-fouling properties. CPs can be easily formed and controlled via electrochemical techniques; however, the main drawback of this type of electrodes concerns the relatively poor sensitivity which could be resolved by using suitable ionophores or CNTs, compared to sensitivities offered by metallic electrodes (Section 3). Another way to enhance selectivity is to directly use biomolecules, known to be extremely specific. To date, among all available biomolecules, enzymes, DNA, peptides or whole cells have been reported for ions sensors.

## 5. Electrodes Modified by Biomolecules

In a biosensor, the sensitive and specific element is biological (e.g., microorganisms, cells or parts of cells, cell receptors, enzymes, nucleic acids, antibodies, peptides, *etc*.). This element must be interfaced with a transducer (in this review, we focus only on electrochemical detectors) that transforms the signal resulting from the interaction of the analyte with the biological element into a current (the interfacing between biomolecules or cells and sensors is a field of growing interest). High selectivity for the analyte, even in a matrix of other chemicals is a key requirement, which is particularly well met by bioreceptors.

Enzymes are characterized by their specific binding capabilities and catalytic activity. Analyte recognition is enabled through two possible mechanisms: conversion of the analyte into a product (although there is no example of HM ions being an enzyme substrate), or enzyme inhibition (a popular approach). With transduction, since enzymes are catalysts, they allow lower limits of detection compared to common binding techniques. As a drawback, enzymes have a limited lifetime.

DNA strands can also act as receptors for HM cations, due to their negative charge. DNA-cations recognition generally proceeds through encaging of the ions in local tertiary structures formed by self-pairing of a single strand. Recently, DNA-zymes (DNA sequences having catalytic properties) were described for HMs’ detection, through similar mechanisms as classical enzymes.

Peptides are amino-acids sequences which, as in the case of DNA, may fold into specific spatial conformations, driven by hydrogen bonding, Van der Waals forces and, effectively, ionic interactions. Therefore, tertiary structures may be formed into which HM cations can bind. Moreover, like DNA, peptides can be synthesized and selected by automated routes in a combinatorial way, and are therefore extremely powerful receptors.

At last, cells may be used as bioreceptors because their metabolism is sensitive to their environment and can be easily monitored, e.g., through their respiration activity (oxygen consumption). They are commonly used to detect similar toxicity parameters. Among cells, microalgae are particularly efficient because their culture is relatively easy.

### 5.1. Enzymes

**Figure 12:**
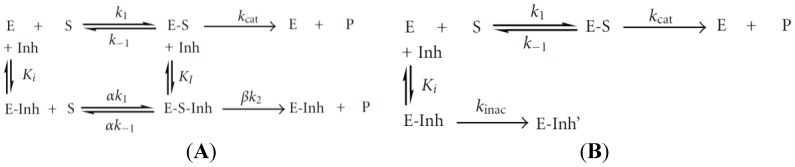
Processes of an enzyme for (**A**) a reversible inactivation or (**B**) an irreversible inactivation. E, enzyme; S, free substrate; P, product; E-S, enzyme-substrate complex; E-Inh, enzyme inhibitor complex; E-S-Inh, ternary complex containing enzyme-substrate inhibitor; K_I_ and K_i_, equilibrium dissociation constants of the E-S-Inh complex and the E-Inh complex, respectively. Reproduced from [102] under Creative Commons Attribution License.

Several enzymes such as acetylcholinesterase and alkaline phosphatase are extremely sensitive to a large number of heavy metals, which act as inhibitors. Enzyme inhibition may be of reversible or irreversible nature (see Figure 12 for the general inhibition mechanisms). For more details, the reader may refer to the review by Turdean [102].

Enzyme products or by-products may be electroactive, meaning their activity may be followed by amperometry. Other enzymes produce or consume protons, meaning their activity can be monitored through pH changes. Other ions may also be produced; in this case, the enzyme’s activity may be monitored through conductimetric measurements.

**Figure 13:**
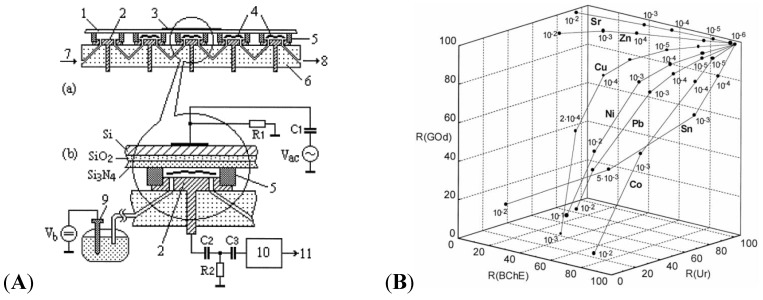
(**A**) 5-channel device with (1) sensitive Si–SiO_2_–Si_3_N_4_ sensors, (2) counter electrode, (4) enzymes, (5) sealing, (6) PMMA body, flow input (7) and output (8); (**B**) Residual activities for urease, BChE and GOD enzymes, respectively, after exposure to selected heavy metal ions at increasing concentrations. Reprinted from [103] with permission from Elsevier.

An excellent example of enzymes for HMs detection was given by Kukla in 1999 [103]. The authors developed a multi-enzyme electrochemical sensor array based on capacitance measurements, illustrated on Figure 13A. Selected enzymes (cholinesterase-BChE, urease-Ur or glucose oxidase-GOD) were immobilized on the array of electrodes, in order to quantify a large number of heavy metal ions among a mixture of these ions, simultaneously. An example of 3D-curves obtained from a three-enzyme device is given on Figure 13B.

In another example, Bagal-Kestwal *et al.* [104] reported an electrochemical biosensor using ultra-microelectrodes modified by invertase and glucose oxidase for the detection of Hg^2+^, Ag^+^, Pb^2+^ and Cd^2+^, with a detection limit of Hg^2+^ around 5 × 10^−10^ M.

However, it is not the objective of this article to review the extensively investigated field of enzyme-inhibition sensors (the reader may refer to comprehensive review focusing on this topic) but to deal with emerging or less investigated receptors such as DNA, peptides, cells or algae.

### 5.2. DNA

DNA probes can be used for the development of HMs sensors as some heavy metals can form complexes with selected nucleic acid bases or structures. Three different strategies can be seen in the literature: coordination between T bases, DNAzymes and G-quadruplex.

#### 5.2.1. (T-Hg^2+^-T) Coordination Based Sensors

**Figure 14:**
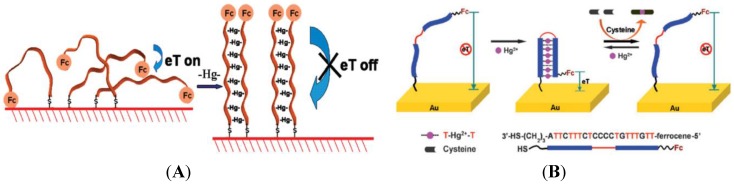
(**A**) Poly-T oligonucleotides tethered on one end on the electrode surface, and labeled on the other end with a ferrocenyl moiety. Upon addition of Hg^2+^, poly-T strands become rigid, drawing Fc away from the surface. Reprinted with permission from [110]. Copyright 2009 American Chemical Society; (**B**) DNA strand carrying a ferrocenyl tag at one end and immobilized on a gold electrode at its other end by a SH group. Under this conformation, the ferrocene is far from the surface and no current flows. A hairpin structure is induced if Hg^2+^ are present, by association between two T bases, which brings the Fc label close to the electrode surface. The sensor can be regenerated by simply unfolding the ferrocene-labeled DNA in 10 µM cysteine. Reproduced from [111] with permission of The Royal Society of Chemistry.

Thymine bases (T) can interact with Hg^2+^ to form T-Hg-T structures which are even more stable than the Watson-Crick adenine-thymine pair [105]. Various electrochemical sensors for Hg^2+^ detection have been proposed using this approach [106,107,108,109]. For example, Liu *et al.* [110] have developed a simple strategy using poly-T oligonucleotides labeled with a ferrocenyl group and immobilized on the electrode surface via self-assembly of the terminal thiol moiety (Figure 14A). In the presence of Hg^2+^, a pair of poly-T oligonucleotides can cooperatively coordinate with Hg^2+^, which triggers a conformational reorganization of the poly-T oligonucleotides from flexible single strands to relatively rigid duplex-like complexes, thus drawing the ferrocenyl tags away from the electrode with a substantial decrease of the redox current.

Han *et al.* [111] have developed an approach using a single-stranded DNA hairpin structure with T-T mismatch, labeled with a ferrocenyl tag and immobilized onto polycrystalline gold surface through self-assembled S-Au bonding. Addition of Hg^2+^ induces conformational change from an open structure to a restricted hairpin structure leading to the increase of the electrochemical signal due to ferrocene (Figure 14B). A detection limit of 0.1 µM was obtained. This system could be reused after addition of 10 µM of cysteine. Zhuang *et al.* [112] used the same kind of strategy but with a hairpin structure labeled with ferrocene in the middle of the loop. Addition of Hg^2+^ opens the hairpin resulting in the ferrocenyl tag close to the electrode to increase in redox current. Detection limit of 2.5 nM was obtained. This system could be reused after addition of iodide. Wu *et al.* [113] obtained a limit of detection of 0.06 nM using an immobilized single-stranded DNA_1_ hybridized with a second DNA_2_ strand labeled with ferrocenyl, which leads to high redox current. In the presence of Hg^2+^, (T-Hg^2+^-T) base pairs induced the folding of the oligonucleotide #1 into a hairpin structure, resulting in the release of the ferrocenyl-tagged oligonucleotide #2 from the electrode surface with a substantially decreased redox current.

#### 5.2.2. DNAzymes-Based Sensors

**Figure 15:**
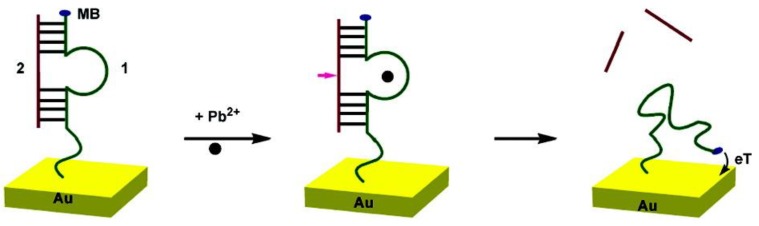
Scheme of the DNAzyme-based electrochemical sensor. Upon addition of Pb^2+^, the DNA_2_ is cleaved, which gives more freedom to MB-labeled DNA_1_. Reprinted with permission from [119]. Copyright 207 American Chemical Society.

**Figure 16:**
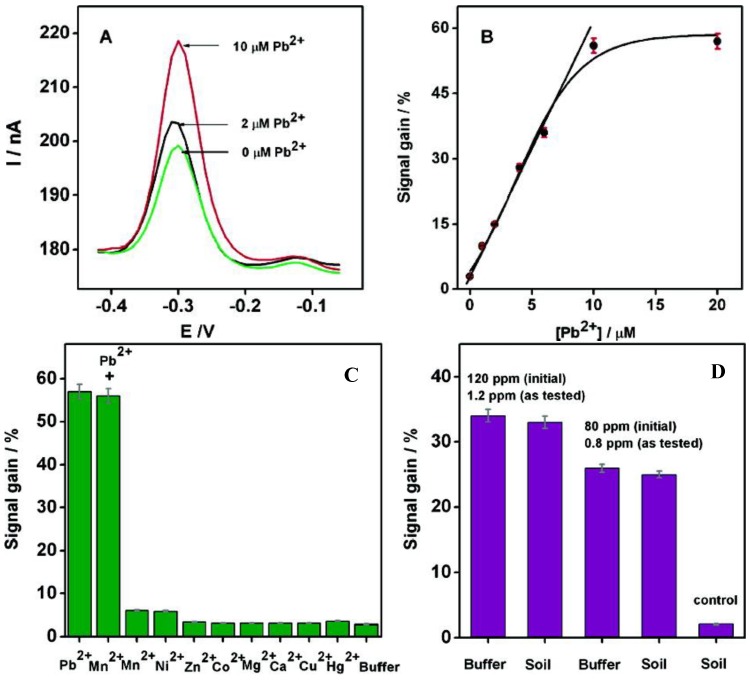
(**A**) Square wave voltammograms after 1 h incubation at increasing concentrations of Pb^2+^; (**B**) Calibration curve; (**C**) Specificity and selectivity experiments; (**D**) Current changes obtained from soil extracts spiked with given lead concentrations, *versus* control. Reprinted with permission from [119]. Copyright 207 American Chemical Society.

DNAzymes are DNA sequences having the property to catalyze specific chemical and biological reactions, such as cleavage of the ribonucleic acid target. Some DNAzymes use divalent metal ion as cofactors; in the presence of this metal ion, the substrate is irreversibly cleaved into two fragments at the cleavage site [114]. Recently, DNAzymes have been used for the detection of lead [115,116,117] and copper [118]. Xiao *et al.* [119] have proposed a very elegant method for lead detection based on the use of a surface-immobilized, methylene-blue modified DNAzyme assembly (Figure 15). Without Pb^2+^, DNAzyme adopts a hairpin structure with the methylene blue away from the surface. In the presence of Pb^2+^, the substrate strand was cleaved. Therefore, the released enzyme strand become more flexible and facilitates the electron transfer between methylene blue and the electrode. The signal is proportional to the concentration of Pb^2+^ and the detection limit is 300 nM (Figure 16A,B).

This sensor was sufficiently specific and selective that it could be used in experiments with complex matrixes such as soil extracts, into which given quantities of Pb^2+^ were added. The results obtained were comparable to those obtained from simpler matrixes such as aqueous buffer (Figure 16C,D).

#### 5.2.3. G-Quadruplex-Based Sensors

DNA G-quadruplex are nucleic acid sequences rich in guanine and capable of forming a four-stranded structure, which is stabilized in the presence of a cation such as K^+^, Na^+^ or Pb^2+^. In 2011, Lin *et al.* [120] proposed an approach using an immobilized unlabeled DNA strand for the detection of Pb^2+^. In the presence of Pb^2+^, the guanine-rich strands switched from a random coil to a quadruplex structure, which was followed by impedance spectroscopy spectroscopy, which lead to a decrease in the charge transfer resistance. A LoD of 0.5 nM was reached. Li *et al.* [121] developed a similar approach using crystal violet as a quadruplex redox binding indicator (crystal violet intercalates into DNA strands). The same approach was used very recently by Jarczewska *et al.* [122] who performed tests in real water, using impedance spectroscopy and methylene blue (MB) as external redox intercalator. Due to the fact that the G-quadruplex induces a folding of the DNA strands, the redox signal coming from MB rises upon addition of Pb^2+^, with a LoD of ca. 50 nM. Without evidencing the same G-quadruplex structure, Lian *et al*. [123] described a differential pulse voltammetry sensor based on the interaction of Pb^2+^ with DNA strands wrapped around single-walled carbon nanotubes (SWNT), with Fe(CN)_6_]^3−/4−^ acting as redox indicator in solution. They demonstrated a LoD of ca. 10^−10^ M in buffer solution (pH 5.0).

### 5.3. Peptides

**Figure 17:**
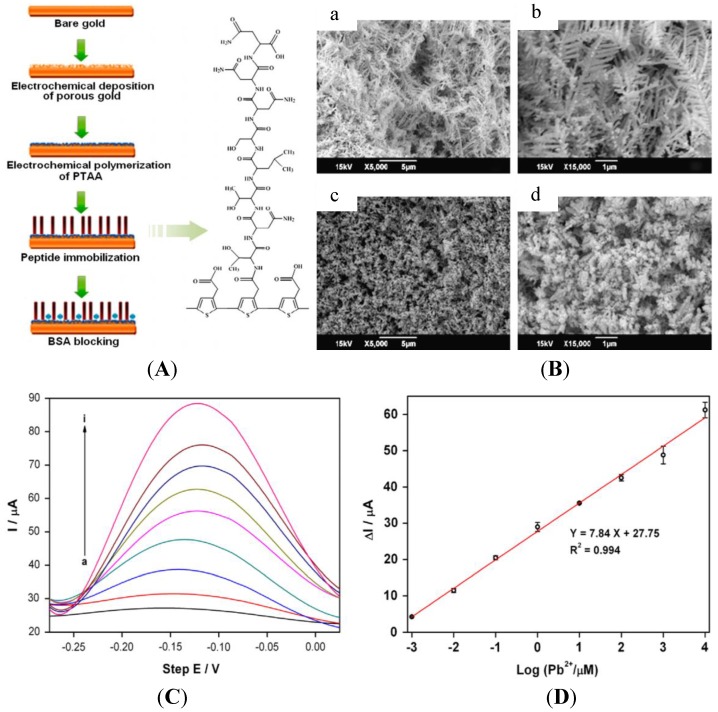
(**A**) Scheme of the procedure for fabricating the peptide modified electrode; (**B**) SEM images of porous gold (a and b) and PTAA-covered porous gold (c and d) layers at different magnifications; (**D**) SWVs with Pb^2+^ concentration from 1 nM–10 mM; (**D**) Calibration curve. Reprinted from [126] with permission from Elsevier.

Peptides and amino acids have been studied for a long time as recognition elements for heavy metal ion detection, in particular for electrochemical sensors [124]. The 20 major amino acids are: alanine (A), Arginine (R), asparagine (N), aspartic acid (D), cysteine (C), glutamic acid (E), glutamine (Q), glycine (G), histidine (H), isoleucine (I), leucine (L), lysine (K), methionine (M), phenylalanine (F), proline (P), serine (S), threonine (T), tryptophan (W), tyrosine (Y) and valine (V). Recent articles reported innovative devices or approaches. For instance, Viguier *et al.* [125] described nanofibrils of four octapeptides (NSGAITIG, NCGAITIG, CNGAITIG, CSGAITIG) having self-assembling properties, which demonstrated recognition capabilities for copper ions. The authors claimed a LoD of ca. 20 μM using cyclic voltammetry, but did not describe the mechanisms involved. Recently, a heptapeptide (TNTLSNN) was identified to selectively bind Pb^2+^ and subsequently immobilized on top of a nanoporous gold electrode [126] through electrodeposition of poly(thiopheneacetic acid) followed by covalent coupling via amide linkage (Figure 17A). After preconcentration by the peptide, Pb^2+^ was detected using ASV, with a LoD of ca. 1 nM (Figure 17D).

**Figure 18:**
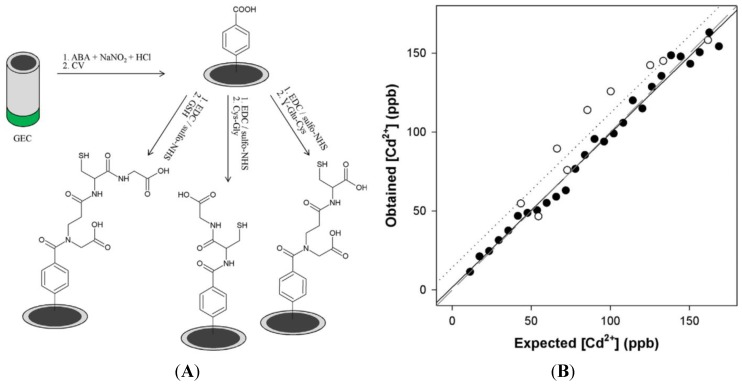
(**A**) GSH, γ-Glu–Cys and Cys–Gly electrografting; (**B**) Measured *vs*. expected concentrations for Cd^2+^. The LoD is below 20 ppb. Reprinted from [127] with permission from Elsevier.

Very recently, Serrano *et al.* [127] reported a three-electrode device in which each electrode was functionalized with a different peptide (glutathione, Cys–Gly and γ-Glu–Cys) through aryl diazonium electrografting. Cd^2+^, Pb^2+^ and Zn^2+^ were determined with this device, using differential pulse adsorptive stripping voltammetry (Figure 18).

A mathematical treatment of the currents measured at each electrode was applied to eliminate interferences, so that simultaneous determination of these three heavy metal ions was achieved. GSH has shown the best sensitivity, followed by Cys–Gly and γ-Glu–Cys.

### 5.4. Whole Cells

Detection of heavy metal ions using whole cells has been described since several decades, mostly using optical transduction techniques such as fluorescence (cells fluoresce in response to a target.). However, these approaches need marking, which has disadvantages, such as complexity and high cost [128]. Living cells are particularly useful for detection of traces of environmentally toxic compounds because these molecules or ions interfere with one or several internal biological processes (e.g., photosynthesis) and produce a modification of the cell’s activity. The major difference between living cells-based biosensors and classical biosensors is the same as between a canary in a coalmine and a methane or carbon monoxide sensor: the sensor will detect only methane or carbon monoxide, but the canary will detect both, often with a better reliability. Similarly, trouts are known to be particularly sensitive to river water pollution, although not necessarily to a single pollutant, and in a physiologically relevant manner [129]. The problems arising with the use of living animals, aside from ethics, are that their interfacing with electronic transducers is particularly unstable, obliterating any quantification possibilities, whilst cells can be immobilized on a surface and used as active sensing elements. From this point of view, owing to its simple instrumentation, high sensitivity and low-cost, electrochemical transduction is particularly pertinent. Indeed, it is relatively easy to immobilize whole cells on electrodes and electrochemically monitor redox species which participate in the cells’ living processes, e.g., oxygen, hydrogen peroxide or protons [130,131,132,133].

**Figure 19:**
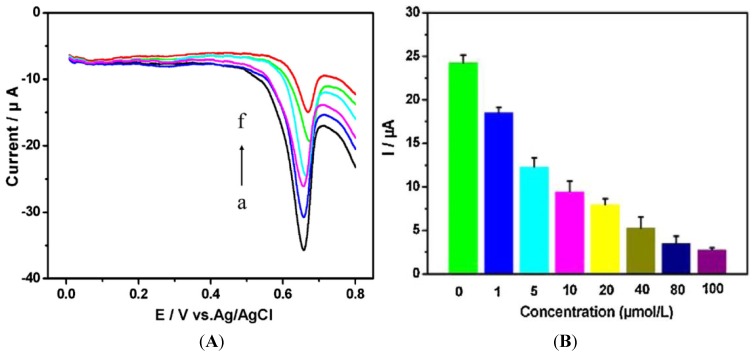
(**A**) CVs of HeLa cell suspension (2.0 × 10^5^ cells·mL^−1^) treated during 30 h with 20 μM of heavy metals solutions. (a) no HM, (b) Zn^2+^, (c) Pb^2+^, (d) Cu^2+^, (e) Cd^2+^, (f) Cr^6+^; (**B**) Peak currents obtained after exposure to different concentrations of Cr^6+^. Reprinted from [135] with permission from Elsevier.

Adam *et al.* [134] have described an electrochemical method for studying the behavior of Escherichia coli that is modified in order to express the human metallothionein gene (MT-3). MT-3 is a cysteine-rich protein known to bind metal ions; after exposure of the bacteria to heavy metal ions, metallothionein was isolated using fast protein liquid chromatography and quantified by electrochemical methods (through typical cysteine oxidation). Its interactions with cadmium and lead ions showed a decline in electrochemistry due to metal ions binding to cysteine. Zhu *et al.* [135] reported a label-free electrochemical method based on the direct voltammetric response of human cervical carcinoma (HeLa) cells on a reduced graphene oxide-modified glassy carbon electrode. Five heavy metals were tested (Cr, Cd, Cu, Pb, Zn) with an excellent correlation using a traditional quantification assay. Actually, the method is cell counting, which is based on the quantity of guanine and xanthine secreted in the cell suspension and oxidized on the electrode, yielding an anodic potential of around +0.67 V *vs*. Ag/AgCl (Figure 19).

**Figure 20:**
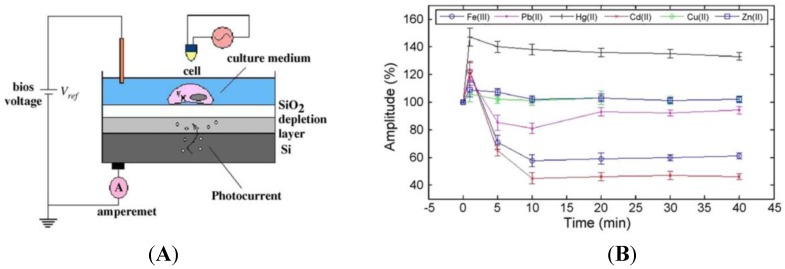
(**A**) Schematic drawing of a LAPS device to monitor beating of cardiomyocytes immobilized on the semiconductor layer. The light pointer is focused above the LAPS, and illuminates vertically above the desired cells. Depending on the bias voltage, photocurrent of the LAPS is monitored by the detection system through peripheral circuit; (**B**) Effect of heavy metals on the spontaneous beating amplitude of the cardiomyocytes recorded by LAPS (mean ± S.E.M.; *n* = 7). Reprinted from [136] with permission from Elsevier.

Liu *et al.* [136] described a novel biosensor for monitoring the changes in electrophysiological activity upon heavy metal exposure. They developed a light-addressable potentiometric sensor (LAPS) [137,138] based on cardiomyocytes immobilized on a semiconductor. Upon illumination of the semiconductor through the cardiomyocytes, the latter produce an ionic current and also change their shape, which lead to a fluctuation of the photocurrent (Figure 20A). After being exposed to different heavy metal ions (Hg^2+^, Pb^2+^, Cd^2+^, Fe^3+^, Cu^2+^, Zn^2+^), cardiomyocytes demonstrated characteristic changes in terms of beating frequency, amplitude and duration under the different toxic effects of ions, in less than 15 min. The limit of detection depends on the metal, but sits between 1 μM and 10 μM (Figure 20B). This technique paves the way to new bio-analytical methods, instrumentation and cell-based assays for *in vitro* toxicity screening.

**Figure 21:**
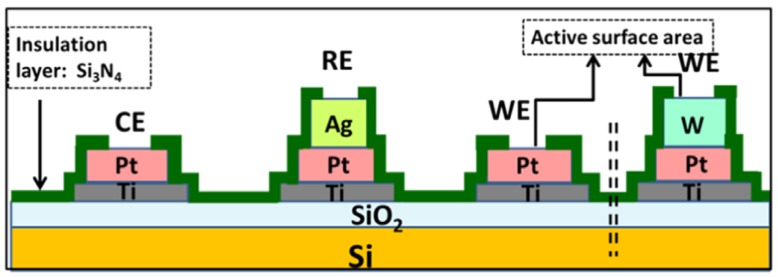
Schematic representation of the micro-fabricated electrochemical sensor. Reprinted from [140] with permission from Elsevier.

Micro-algae such as chlorophyta, cyanobacteria and diatoms are also very sensitive to changes in their environment, enabling the detection of traces of pollutants [139]. Tsopela *et al.* [140] described a microfabricated electrochemical biosensor (Figure 21) containing three types of electrode materials (Pt black, Pt/IrO_2_ and W/WO_3_), which are able to detect O_2_, H_2_O_2_ and pH changes from the photosynthetic and metabolic activities of algae.

**Figure 22:**
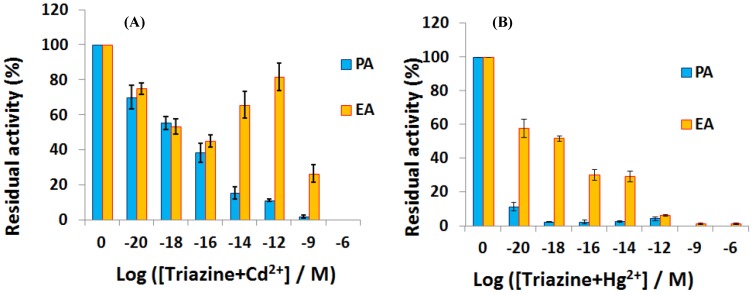
Residual activity of phosphatases and esterases enzymes after 12 h exposure to a couple of organic pollutant and heavy metal ion such as (**A**) (triazine + Cd^2+^) and (**B**) (triazine + Hg^2+^). Reproduced from [143] with permissions.

Chouteau *et al.* [141] described interdigitated conductimetric electrodes onto which the microalgae Chlorella vulgaris is immobilized. This microalgae contain alkaline phosphatase which locally produces phosphate ions, modifying the local ionic conductivity. Alkaline phosphatase is known to be inhibited by heavy metals such as Cd^2+^ and Zn^2+^, with a LoD of ca. 10 ppb for a 30 min long exposure. A similar sensor was described later by the same research team [142] where the microalgae were immobilized on the interdigitated area through self-assembled monolayers (SAMs) of alkanethiol. Very recently, using similar interdigitated electrodes, Tekaya *et al*. [143] described a bi-enzymatic biosensor made by immobilizing Arthrospira platensis cells and using impedance spectroscopy to characterize cells’ activity. Phosphatase and esterase activities were inhibited, respectively, by heavy metals such as Cd^2+^ and Hg^2+^, and by selected pesticides, with extremely low LoD of ca. 10^−20^ M. Complex matrixes such as a municipal wastewater were used to demonstrate its pertinence (Figure 22).

## 6. Conclusions and Perspectives

There is an important bibliography concerning electrochemical detection of heavy metals, most of them based on stripping voltammetry, using various materials for modification of the electrodes. Some of these sensors are very sensitive, some are very selective and some are both sensitive and selective. However, none have replaced current standard analytical procedures. The main advantages and drawbacks of the major techniques reviewed in this manuscript are briefly summarized in Table 3.

As described in Section 5.4, the use of living materials is the classical technique to detect pollutants, and biomaterials such as whole cells or enzymes are certainly the most promising sensing elements for interfacing with modern electronics. Electrochemical transistors are the most compatible with biointerfaces [144] because they are sensitive to both ions and electrons. For example, a conducting polymer-based transistor was very recently described for monitoring the activity of living epithelial cells, with a proof-of-concept for toxicology studies and diagnostic applications [145]. Another group described an organic electrochemical transistor (OECT) based on poly(3,4-ethylenedioxythiophene):poly(styrene sulfonic acid) (PEDOT:PSS) successfully used for the detection of marine diatoms (algae) directly in seawater. These living microalgae, when immobilized at the gate electrode, produce a gate offset voltage of several tens of mV, which significantly shifts the transfer curves of the OECT to higher gate voltage. Such organic transistors are perfectly able to monitor the activity of these microalgae, known to be sensitive to heavy metals. They pave the way for biochemical sensing, particularly in marine environments [146]. Still, with the same kind of OECT, Kergoat *et al.* demonstrated the possibility to functionalize PEDOT:PSS with platinum NPs in order to complement the best sensitivity an enzymatic activity. They made the proof of concept with AChE for a completely different application, but as AChE is known to be particularly inhibited by HMs, this work also paves the way to analytical applications [147]. Lastly, even though they are not organic transistors, electrolyte-gated transistors (EGTs) based on ZnO thin film were used for physiological and environmental monitoring, with proof of concepts for enzymatic glucose sensing and also selective ion sensing [148].

**Table 3:** Mainadvantages and drawbacks of the major techniques reviewed in this manuscript.

Techniques	Advantages	Drawbacks
Metallic or carbon electrodes	Sub-ppb detection Easily miniaturisable On-field detection	Lack of specificity Lack of reproducibility Formation of intermetallic compounds Formation of biofilms and fouling
Chemically modified electrodes with CPs	Sub-ppb detection Specificity Anti-fouling	Sensibility Stability
Electrodes modified with biomolecules	Specificity	Costs Long-term stability Not yet available for on-field detection

Interfacing between electrodes and biomolecules is still a work in progress. To aid in this development, the user interface must progress as well. Very recently, a paper described a handheld device that couples an electrochemical sensor (illustrated for analysis of lead, cadmium, and zinc in water) with a mobile phone capable of supplying power and data transmission, for in-field environmental monitoring, even with a low level of technical expertise [149]. Clearly, there is plenty of room for smart bioelectrochemical sensing of HMs in the years to come.
